# Effects of Music Therapy on Drug Therapy of Adult Psychiatric Outpatients: A Pilot Randomized Controlled Study

**DOI:** 10.3389/fpsyg.2016.01518

**Published:** 2016-10-07

**Authors:** Mario Degli Stefani, Michele Biasutti

**Affiliations:** ^1^Department of Mental Health, 2° Servizio Psichiatrico Ulss 16 Padova, PadovaItaly; ^2^Department of Philosophy, Sociology, Education and Applied Psychology, University of Padova, PadovaItaly

**Keywords:** music therapy, drug treatments, adult psychiatric outpatients, neuroleptics, antidepressants

## Abstract

**Objective:** Framed in the patients’ engagement perspective, the current study aims to determine the effects of group music therapy in addition to drug care in comparison with drug care in addition to other non-expressive group activities in the treatment of psychiatric outpatients.

**Method:** Participants (*n* = 27) with ICD-10 diagnoses of F20 (schizophrenia), F25 (schizoaffective disorders), F31 (bipolar affective disorder), F32 (depressive episode), and F60 (specific personality disorders) were randomized to receive group music therapy plus standard care (48 weekly sessions of 2 h) or standard care only. The clinical measures included dosages of neuroleptics, benzodiazepines, mood stabilizers, and antidepressants.

**Results:** The participants who received group music therapy demonstrated greater improvement in drug dosage with respect to neuroleptics than those who did not receive group music therapy. Antidepressants had an increment for both groups that was significant only for the control group. Benzodiazepines and mood stabilizers did not show any significant change in either group.

**Conclusion:** Group music therapy combined with standard drug care was effective for controlling neuroleptic drug dosages in adult psychiatric outpatients who received group music therapy. We discussed the likely applications of group music therapy in psychiatry and the possible contribution of music therapy in improving the psychopathological condition of adult outpatients. In addition, the implications for the patient-centered perspective were also discussed.

## Introduction

It is well known that music is connected to mood and that a certain piece of music can make people feel blessed, sad, lively, or relaxed (Biasutti, 2015a). The effects of music on a person’s mindset and well-being are evident, and music has been used in various settings and conditions to control and improve health conditions (Biasutti and Concina, 2013). Music has been studied in several therapeutic settings for managing psychological conditions such as anxiety and stress, and there is strong scientific evidence supporting these positive effects. Music therapy treatments are categorized as expressive therapies and non-verbal techniques for facilitating, expanding and shaping patients’ expression and communication modes (Manarolo, 2005). The improvement of these skills provide patients with a comfort zone in which to express themselves while developing confidence and self-efficacy.

Music therapy treatment may help in shifting from a disease-centered perspective to a patient-centered perspective in the psychiatric setting and in determining new dynamics in the care relationship. Music therapy may facilitate a patient-centered perspective with an approach based on creativity and personal empowerment rather than external control of symptoms, as is the case with drug control.

A patients’ engagement in healthcare is crucial and is widely recognized as a critical component of a high-quality health care system (Mead and Bower, 2000; Coulter and Ellins, 2007; Coulter, 2012; Graffigna et al., 2015). Several health care institutions at the forefront of policies support the promotion of a patient-centered perspective (Hibbard et al., 2004; Barello et al., 2012). The consumer-centered mental health framework could be used for finding alternative solutions to complement drug usage in order to improve the symptoms of patients and thus improve the participation and effective collaboration of patients in the management of their care (Lawn et al., 2007; Kreyenbuhl et al., 2009; Kukla et al., 2013).

There are several advantages for the use of music therapy in mental health care that can facilitate the shift from a disease-centered perspective to a patient-centered perspective. Compared with drug therapies, music therapy can help to improve patients’ symptoms and quality of life in alternative and more holistic ways. This approach can lead to patients more effectively engaging in their care management, consequently resulting in improved health and well-being outcomes, including such effects as positive recovery attitudes (Loh et al., 2007; Patel et al., 2008; Livingston et al., 2013).

### Background

The current study is focused on how a patient-centered perspective could be applied in psychiatry using music therapy as a rehabilitation technique. There are many studies in the literature that have focused on the effects of music therapy in such settings as psychiatry. Several reviews and meta-analyses have shown that music therapy, in combination with drug treatments, has significant effects on the positive and negative symptoms of psychosis, depression and the well-being of affected individuals (Maratos et al., 2008; Gold et al., 2009; Carr et al., 2013). The positive effects of music therapy have also been observed in the pathology of schizophrenia (Yang et al., 1998; Talwar et al., 2006). Jung and Newton (2009) performed a comparative analysis of the existing literature using the Cochrane database to explore the effects of 28 alternative therapies for schizophrenia, psychosis, and bipolar disorder. They noted that music therapy is one of the four most effective interventions in psychiatry. Other research has considered the effectiveness of music therapy on patients’ quality of life and spirituality (Grocke et al., 2014), among schizophrenic in-patients needing acute care to reduce negative symptoms and improve interpersonal contact (Ulrich et al., 2007), and for reducing patients’ depression (Erkkilä et al., 2011).

The issue of the reduction of drug use by mental health patients as an effect of the outcomes of music therapy sessions (which is the subject of the current research) has been regarded with less interest by researchers, likely because of the difficulty of accessing such data and the complexity of data retrieval. Changes in drug treatments are often evaluated secondarily without being deemed a fundamental aspect of research. Chen et al. (2014) compared the drug dosages of patients in two groups (experimental and control) and found similarities in drug intake between the two groups. However, we argue that drug dosage should be considered a fundamental variable of research because it has a significant impact on disease management and because it involves a number of obvious advantages in an individual’s treatment.

The literature also discusses how music therapy techniques can become complementary or adjuvant to drug therapy and how music therapy can provide psychological support with the consequent need to carefully consider the scientific evidence of the effectiveness of music therapy. In a meta-analysis of five studies, Maratos et al. (2008) compared the efficacy of music therapy to other therapies, such as drug and psychological support, and found that in four of the five studies analyzed, a greater reduction in symptoms of depression was reported among randomized patients after music therapy compared to those who received standard care. Similar results have also been highlighted by Erkkilä et al. (2011). Other studies have shown that in various types of patients, the therapeutic value of music can lead to a reduction of the anxiety state and a consequent decrease of drugs for the control of anxiety (Lepage et al., 2001). Lepage et al. (2001) found that the use of music resulted in decreased use of sedatives in interventions involving spinal anesthesia.

Few studies have examined changes in drug treatment as a result of music therapy interventions. Morgan et al. (2011) considered variations in drug treatment in patients between the ages of 17 and 55 years with a diagnosis of schizophrenia, schizoaffective disorder, and bipolar affective disorder with a framework of psychotic symptoms in the acute phase after participating in music therapy sessions. The patients were divided into two randomized groups: the experimental group, which participated in four sessions of individual active music therapy, and the control group, which participated in four sessions of music listening. Several scales have been proposed for patients before and after treatment, such as the Brief Psychiatric Rating Scale and the Calgary Interview Guide for Depression, and the average weekly patient dose of drugs, such as antipsychotics, mood stabilizers, benzodiazepines and antidepressants, has been monitored. There were no significant differences in drug dosage before and after music therapy treatments, whereas significant results were found using the Brief Psychiatric Rating Scale. The outcomes of this research are likely to be related to the short duration of music therapy treatment.

### Literature Review Summary

The background analysis showed the effectiveness of music therapy treatments in psychiatry and suggested that music therapy may provide a means of improving mental health among psychiatric patients (Yang et al., 1998; Talwar et al., 2006; Maratos et al., 2008; Gold et al., 2009; Jung and Newton, 2009; Carr et al., 2013). However, its effects on drug therapy have not been deeply explored, and no significant reduction in the drug treatment of patients following music therapy treatments has been shown (Morgan et al., 2011). The results of the research by Morgan et al. (2011) were likely influenced by the limited number of music therapy sessions (only four). Few studies have correlated the effects of music therapy with drug dosage in the treatment of psychiatric patients by considering these variables fundamental in the formulation of the experimental hypotheses. The need for better methodological control and conditions with a specific focus on the music therapy approach has also been noted.

### Aim of the Study and Research Question

The current research aimed to fill the gaps of previous research by examining how prolonged group music therapy may affect drug assumption in adult psychiatric outpatients in a controlled trial. The research methodology adopted a quantitative approach using an experimental design that included an experimental group (MT) and a control group (CTR). The MT group participated in group music therapy sessions, whereas the CTR group experienced other non-expressive group activities (psycho-educational group activities on well-being). The research resulted in an analysis of drug dosages for all of the patients by consulting their medical records. The pharmacological treatments were classified according to the following four primary categories: neuroleptics, benzodiazepines, mood stabilizers, and antidepressant medications. The following research question was considered: does participation in group music therapy sessions affect drug dosage in adult outpatients with serious psychiatric problems?

## Materials and Methods

### Participants

The sample consisted of 27 psychiatric outpatients ranging in age from 27 to 57 years (average age, 42 years) who were followed at an outpatient Mental Health Centre (MHC) in the northeast region of Italy. Participants were recruited by considering all the patients under the care of the MHC. The inclusion criteria required that the participants’ primary diagnoses were F20 (schizophrenia), F25 (schizoaffective disorders), F31 (bipolar affective disorder), F32 (depressive episode), and F60 (specific personality disorders) according to the ICD-10 classification. These inclusion criteria were determined by the need to focus the research on psychiatric disease. Other types of diseases were not considered, such as drinking problems, post-traumatic stress disorder, and substance misuse disorder. Additionally, the outpatients were required to have pharmacological treatments in the following four primary drug categories: neuroleptics, benzodiazepines, mood stabilizers, and antidepressant medication. Fourteen participants were assigned to the MT group, and 13 were randomly assigned to the CTR group. Only the MT group participated in the group music therapy activities, whereas the CTR group experienced other non-expressive group activities (psycho-educational group activities on well-being).

**FIGURE 1:**
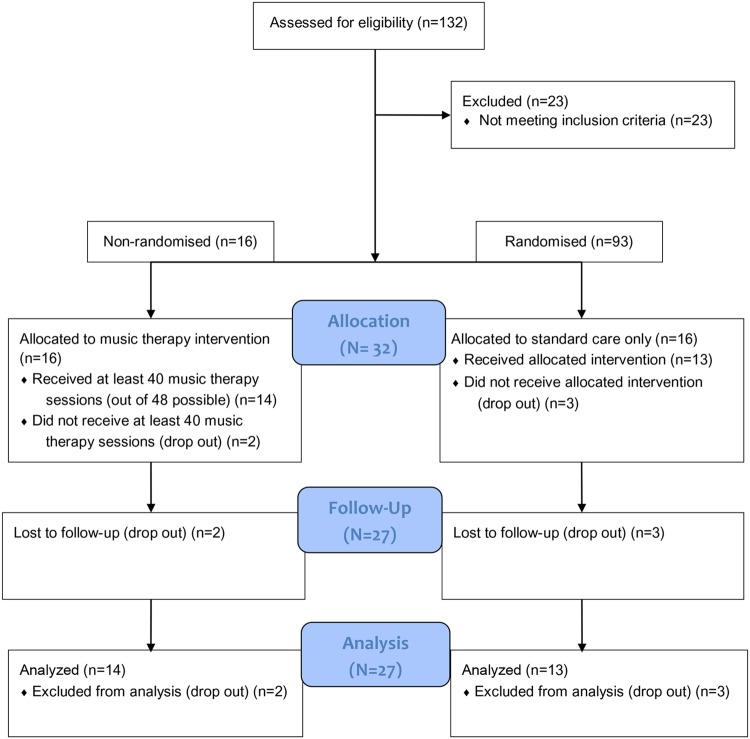
**Flow chart of the referral process**.

We assessed 132 participants for eligibility. Of this group, 23 were deleted because they did not meet the inclusion criteria. Of the remaining 109 participants (132 – 23 = 109), 16 participants were assigned to the music therapy activities. Of the remaining 93 subjects (109 – 16 = 93), a group of 16 participants was randomly extracted for the control group. The process of random assignment was performed by an independent collaborator who was not responsible for determining the eligibility of the patients. The random assignment was determined with a random number generator with the use of sequentially numbered, opaque, sealed envelopes (SNOSE). A flow chart of the referral process is presented in **Figure 1**.

The participants had been admitted to the MHC in a day-care community. Their medical records had been established for at least 6 years. They were not in an acute or subacute state, and they had no hospitalisations in the previous 6 months. The participants of the two groups had similar characteristics with regard to age, gender, prognostic factors, and psychiatric disease. The age of the 14 psychiatric outpatients of the MT group ranged from 29 to 57 years (average age of 43 years), and the group was composed of seven males and seven females. The age of the 13 psychiatric outpatients of the CTR group ranged from 27 to 55 years (average age of 41 years), and the group was composed of five males and eight females. A group comparison using the χ^2^ test showed no significant differences between the MT group and the CTR group with regard to age, gender, and psychiatric disease.

The psychiatric participants were diagnosed with psychosis, bipolar disorder, and/or borderline personality disorder, and all displayed psychotic features. The patients involved in the study were considered serious with regard to psychosis. The participants were independent and autonomous. The majority of them lived with their family of origin. Of the participants, 63% were men, and 22% were married and living with their spouse, whereas only one was living in a community apartment. Regarding socio-cultural status, 14% had a university degree, and 37% had a high school diploma, and only 37% had a job.

### Music Therapy Activities

The music therapy activities were conducted in group sessions of 2 h each on a weekly basis throughout 1 year for a total of 48 sessions. The music therapy activities were based on active techniques in which the participants were asked to play and improvise. Improvisation was based on the processes outlined by Biasutti and Frezza (2009) and Biasutti (2015b). The purpose of the group music therapy activities was to constitute a stable human therapeutic frame capable of supporting the relevant manifestations of suffering expressed by the outpatients. “The therapeutic process is based on the mutual construction of meaning of emerging thoughts, images, emotional content, and expressive qualities that often originate from the musical experience and are then conceptualized and further processed in the verbal domain” (Erkkilä et al., 2011, p. 134). The setting consisted of chairs arranged in a circle with the instruments (e.g., djembe, darbuka, cymbals, wood blocks, rattles, bongos, and maracas) placed in the center.

The music therapy team, led by a music therapist, included a psychiatrist-psicotherapist, and a nurse. In addition to these professionals, a volunteer psychologist and a music therapist trainee attended the sessions. The large number of operators was necessary for the validity of the setting and is a constitutive feature of group music therapy activities (Manarolo, 2006). All of the members of the music therapy team participated in the group sessions and were involved in the musical productions. At the end of each session, a reflective discussion was scheduled in which the participants had the opportunity to express the feelings they experienced during the group music therapy session. Moreover, there was a monthly meeting of the music therapy team for sharing and discussing the progress of the sessions. The 14 participants in the MT group were divided into two groups of eight and six participants who had separate group sessions to ensure a lower dispersion and greater attention by the music therapy team. The two groups were conducted by the same music therapy team using the same music therapy methodology. These groups were designed for a maximum of 10 participants (Manarolo, 2006).

### Procedure

The data were collected and the patients’ medical records over a 1-year period were analyzed to extrapolate the historical drug dosage for each patient. The data collection transpired over a sufficiently long period of time to appreciate the general course of drug therapy. Pre- and post-music therapy periods were distinguished. The pre-music therapy period was when the patients did not attend the music therapy sessions. This period was useful for collecting baseline data on drug dosage. The post-music therapy period was after the end of the music therapy sessions. The patients participated in an ongoing music therapy activity for a year, which is considered the minimum duration for detecting significant results of music therapy (Gold et al., 2009). Before starting and at the end of the activities, the dosages of the drugs taken by the patients on a daily basis were noted, and the total quantity of drugs consumed in a month was calculated. Medications were controlled by psychiatrists, who determined whether doses should be increased or decreased. Medications were controlled every 2 weeks, and this control was independent of the music therapy sessions offered. The psychiatrists who controlled the drug dosage of the participants were blind to the research procedures and to the participants’ allocation, and they had no role in the research.

With regard to drugs, the participants were administered a variety of drugs summarized into 35 types, which are placed in the following four broad categories: neuroleptics, benzodiazepines, mood stabilizers, and antidepressants. To obtain standardization of the data, the dosage was reported in Mg and was later converted into a percentage by dividing the daily dose into the maximum dosage per day. The mean daily dose for the long-acting depot drugs, which were taken every 2/4 weeks, was also calculated. Additionally, the adult outpatients participated in monthly individual monitoring visits in which their clinical picture was verified. The data from these visits were used to triangulate the results.

This study was conducted in accordance with the recommendations of the British Psychological Society with informed consent from all subjects. All subjects gave informed consent in accordance with the Declaration of Helsinki. For medical record consultations, the director of the MHC obtained the proper authorisation.

## Results

### Methods for Data Analysis

The data collected included the changes over time of drug treatments for the four types of drugs during pre- and post-conditions. For data analysis, the statistical program SPSS 22 was used. The descriptive statistics were computed, and the Kolmogorov–Smirnov normality test was conducted to verify whether the distributions of the considered pharmacological groups were normal. The results showed that all of the categories met the criteria of normality, with the exception of mood stabilizers at *p* < 0.05. For this category, the non-parametric Friedman Test was performed, which allowed us to separately isolate the time effect within the groups. To calculate the other drug categories, a paired *t*-test was used to compare the MT and CTR groups in the pre- and post-conditions. In addition, a group comparison of the drug dosages at baseline was performed, and no group differences were found.

### Drug Treatment

#### Neuroleptics

**FIGURE 2:**
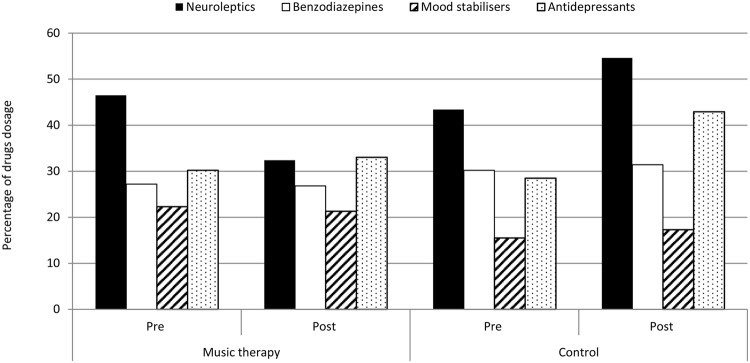
**Changes from baseline (PRE) to post-intervention (POST) in the dosage of the four drug categories for the MT group (*n* = 14) and the CTR group (*n* = 13)**.

Changes in the monthly dosage of neuroleptics for the MT group and the CTR group in the month before the commencement of music therapy and after the group music therapy intervention are shown in **Figure 2**. The two groups had different trends over time with regard to the doses of neuroleptics: the MT group decreased, whereas the CTR group increased. Paired *t*-tests showed that these changes were significant in both groups: the MT group with *t* = 2.28, df = 13, *p <* 0.040, and the CRT group with *t* = -2.32, df = 12, *p <* 0.039.

#### Benzodiazepines

Changes in the monthly dosage of benzodiazepines for the MT group and the CTR group in the month before the commencement of group music therapy and after the music therapy intervention are shown in **Figure 2**. There was no significant difference (paired *t*-test, *p* > 0.05) between the MT group and the CTR group. The benzodiazepines exhibited a similar trend in both groups, and the drug dosage appeared to be stable over time.

#### Mood stabilizers

Changes in the monthly dosage of mood stabilizers for the MT group and the CTR group in the month before the commencement of music therapy and after the group music therapy intervention are shown in **Figure 2**. The non-parametric Friedman test was computed for the mood stabilizers, which showed no significant differences in drug dosage over the two periods for the MT and CTR groups.

#### Antidepressants

Changes in the monthly dosage of antidepressants for the MT group and the CTR group in the month before the commencement of music therapy and after the group music therapy intervention are shown in **Figure 2**. The drug dosage displayed a similar trend in both groups, but the CTR group increased more than the MT group. This difference is evident in the paired *t*-tests, which showed a significant change only for CRT group with *t* = -2.56, df = 12, *p <* 0.025, whereas the MT group reported no significant difference (paired *t*-test, *p* > 0.05).

## Discussion

The present study evaluated the effects of a music therapy intervention on drug dosages in adult psychiatric outpatients. Doses of neuroleptics decreased significantly in the MT group and increased significantly in the CTR group. Doses of antidepressants did not change significantly in the MT group but increased significantly in the CTR group. In contrast, benzodiazepines and mood stabilizers did not demonstrate significant changes in either group. The adult outpatients participated in monthly individual monitoring visits in which their clinical picture was verified. The data from these visits showed an improvement or no worsening of their clinical picture during the group music therapy activities, confirming the results of the diminished drug dosage of neuroleptics. Neuroleptics are an essential drug therapy for psychiatric patients who display symptoms of psychosis, and a significant difference between the MT and the CTR groups emerged. The dosage in the MT group tended to decrease significantly, whereas the dosage in the CTR group increased significantly. The results provide evidence that a group music therapy treatment in combination with standard care, including pharmacological treatment, can affect the dose of neuroleptics (reducing or stabilizing their consumption) and can enhance the improvement of the clinical picture. The data in the present study are in contrast to the findings by Morgan et al. (2011), who reported no significant differences in the dosage of neuroleptics between the experimental and control groups. These differences likely relate to the different number of music therapy sessions in the study by Morgan et al. (2011) compared to the present research. A more extensive explanation is due to the nature of neuroleptics that are used in the treatment of schizophrenia and in diseases such as bipolar disorder, depressive episodes with psychotic onset, and personality disorders. These drugs act to reduce positive symptoms, such as hallucinations, deliria, aggressiveness, and excitement, and have less direct effects on negative symptoms, such as ideational impoverishment, autism, depression, and withdrawal into oneself. Active music therapy plays an important role in reducing the symptoms of psychosis, such as auditory hallucinations, deliria and psychomotor agitation, schizophrenia, and schizoaffective disorders, in patients suffering major depressive disorders. Research has analyzed patients with diagnoses similar to those who participated in this study. It could be argued that group music therapy may have induced the attenuation of positive symptoms as well as negative ones, favoring the likelihood of reduction in the daily dose of the drug.

Regarding benzodiazepines, no significant differences were found for the MT and CTR groups. This observation likely depends on the characteristics of the drug. Benzodiazepines are used as muscle relaxants and are effective for treating symptoms such as anxiety and agitation. They are used for anxiety disorders and depression as well as psychotic disorders, such as schizophrenia. The literature emphasizes that treatment using benzodiazepines should be cyclical; the dose cannot be increased for a period of time, and the drug is typically administered continuously for a relatively short period (Dell’osso and Lader, 2013).

The increase in antidepressant therapy corresponds to the reduction of secondary neuroleptic therapy and to the greater stability of the basis of the psychotic framework. This is a fact that might suggest a maturing capacity of introspection and insight in patients who are able to address greater awareness of the disease and related depressive experiences. This hypothesis could also be related to the induction of more anxious aspects and to the use of benzodiazepines.

With regard to mood stabilizers, the data did not respond to the normality criteria and were subjected to non-parametric tests that showed no significant differences for the MT and CTR groups. It is useful to remember that the changes in dosages for this type of drug are usually less “pointed” than the other categories evaluated because the mechanism of action requires a long time to achieve a therapeutic effect.

These results provide evidence that music therapy can stabilize or reduce the daily amount of drugs administered to psychiatric patients. This observation contrasts with the outcome of Morgan et al. (2011), who did not find significant differences between groups for mood stabilizers.

The other aspects that may have contributed to the positive results of this study include the number of sessions, the music therapy technique and the reflective discussion at the end of the session. The subjects attended 48 sessions within a year lasting 2 h each, which is a suitable number according to the literature that suggests that 16 to 51 sessions are adequate to obtain a positive result (Gold et al., 2009). The music therapy technique was an active approach (i.e., the patients were asked to actively participate in the music performance) and has been considered the most significant means for treating severely ill psychiatric patients (Manarolo, 2005). These methods are considered effective because patients have the opportunity to express feelings that they would not be able to explain in any other way. Another factor is the reflective discussion employed at the end of the music therapy sessions, which allows patients to think about the content that emerged during the sessions and share their thoughts with the group (Bruscia, 1998; Erkkilä et al., 2011). The discussion is a crucial aspect in which the effects of the sound-musical element allow the patient to express or verbalize latent content of which the patient is unaware. This phase, which is considered critical and therapeutic, is supported by the psychiatrist and the entire music therapy team who act as a container for the patient’s emotional reactions.

## Conclusion and Research Perspectives

The results of this research have demonstrated the long-term effectiveness of group music therapy activities with adult outpatients with serious psychiatric issues. In this research, the most relevant analyzed drugs were neuroleptics and antidepressants. These drugs are more potent than other drugs, both in terms of their effects and their side effects. The remaining two types of drugs, mood stabilizers and benzodiazepines, have different characteristics and are not indicative of psychotic pathology. Psychotic drugs are crucial in the treatment course of a psychiatric condition; however, they have several side effects that reduce the patient’s quality of life physically, such as tremors, fatigue, and drowsiness, and socially, such as flattened emotional and social spheres, decreased motivation in participating in daily activities, and a sense of isolation. Group music therapy was effective in controlling psychotic symptoms and successfully reducing the dosage of neuroleptics, thus indicating greater stability in the patient’s clinical picture, which was a significant achievement. The music therapy intervention has the advantage of no side effects and may be beneficial to improving patients’ quality of life (Grocke et al., 2014) by reducing the drug dosage.

The data presented in this study are encouraging considering that the patients’ characteristics are similar to the clinical picture of severe psychiatric issues. For these patients, continuity in attendance at music therapy sessions is already a significant indicator of success because there was a sharp drop-out in participation for other activities proposed by the MHC of the current research. This research should be considered a pilot study with a limited number of patients. Several limitations could be discussed, such as the low sample size and the measurement of the psychophysical conditions of the outpatients. The data are significant and provide ideas for the continuation of research; however, the data should be validated with a larger group of subjects before generalization. In addition, a set of tools could be associated to measure the enhancement of the psychophysical conditions of the outpatients. The data could be verified in additional research with regard to whether these activities might induce persistent and meaningful effects in combination with drug treatments and how music therapy might limit drug usage. Other aspects that could be explored in future trials include a better understanding of the relationship between the patient and the therapeutic process in relation to individual diseases and the different contexts in which the patient is placed.

### Implications for the Patient-Centered Perspective

The results of the current research could also be discussed in the framework of the patient-centered perspective considering two main points: (1) the potential role of music therapy in providing care that is more holistic and aligned with a patient-centered perspective; and (2) the potential virtuous cycle of symptom reduction through music therapy.

Considering point one, the positive benefits of music therapy in improving symptoms and reducing drug usage in light of the introduction of alternative treatments should be considered. These treatments can better sustain the quality of life of patients and add to their resources for improving symptoms. Furthermore, the findings of the current research provided evidence of the importance of reducing drug usage through alternative methods, such as music therapy, in mental health care. This is an example of how to promote partnerships with patients as well as patient engagement in their care management (Lawn et al., 2007; Kreyenbuhl et al., 2009). In addition, the contribution of music therapy in providing a more holistic and broader approach to mental health care and consequently ensuring that mental health care supports a consumer-centered perspective could be discussed (Lawn et al., 2007; Kukla et al., 2013; Livingston et al., 2013). In this framework, mental disorders are considered not only as something isolated to treat but also as an expression of the individuality of the person (Loh et al., 2007; Patel et al., 2008). Moreover, a bio-psychosocial approach could be adopted, which proposes that symptoms should be treated considering the biological, psychological, and cultural backgrounds of each patient.

Regarding point two, the potential virtuous cycle of symptom reduction through music therapy could be highlighted. In this case, the reduction of drug use induces an improvement in patient engagement with an influence on the general well-being of the person. This cycle could induce a deeper engagement of patients in their care management and therefore exert an influence on such aspects as positive recovery attitudes, higher levels of hope, and fewer symptoms of emotional discomfort (Loh et al., 2007; Kreyenbuhl et al., 2009; Kukla et al., 2013; Livingston et al., 2013). In this framework, music therapy in addition to drug treatment can help in shifting from a disease-centered perspective to a patient-centered perspective in the psychiatric setting to establish new dynamics in the care relationship (Degli Stefani and Xodo, 2015).

## Author Contributions

MDS: Supervised the clinical setting, implemented the music therapy activities, and collected the data. MB: Revised critically the literature, analyzed the data, discussed the results, and wrote the whole paper.

## Conflict of Interest Statement

The authors declare that the research was conducted in the absence of any commercial or financial relationships that could be construed as a potential conflict of interest. The reviewer JM and handling Editor declared their shared affiliation, and the handling Editor states that the process nevertheless met the standards of a fair and objective review.
